# Vertically Processed GaInP/InP Tandem-Junction Nanowire Solar Cells

**DOI:** 10.1021/acsanm.3c05909

**Published:** 2024-01-08

**Authors:** David Alcer, Matteo Tirrito, Lukas Hrachowina, Magnus T. Borgström

**Affiliations:** NanoLund and Division of Solid State Physics, Lund University, Box 118, Lund 221 00, Sweden

**Keywords:** nanowire solar cell, GaInP, InP, tandem junction, photovoltaics

## Abstract

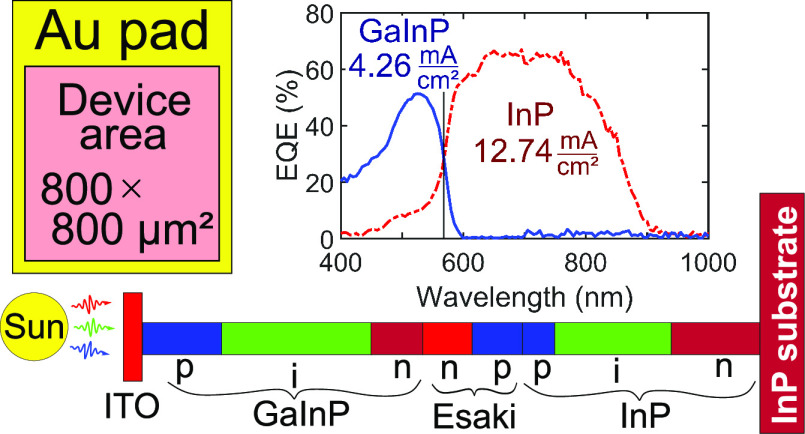

We present vertically processed photovoltaic devices based on GaInP/InP tandem-junction III–V nanowires (NWs), contacting approximately 3 million NWs in parallel for each device. The GaInP and InP subcells as well as the connecting Esaki tunnel diode are all realized within the same NW. By processing GaInP/InP tandem-junction NW solar cells with varying compositions of the top junction GaInP material, we investigate the impact of the GaInP composition on the device performance. External quantum efficiency (EQE) measurements on devices with varying GaInP composition provide insights into the performance of the respective subcells, revealing that the GaInP subcell is current-limiting for all devices. *I*–*V* measurements under AM1.5G illumination confirm voltage addition of the subcells, resulting in an open-circuit voltage of up to 1.91 V. However, the short-circuit current density is low, ranging between 0.24 and 3.44 mA/cm^2^, which leads to a resulting solar conversion efficiency of up to 3.60%. Our work shows a path forward toward high-efficiency NW photovoltaics and identifies critical issues that need improvement.

## Introduction

III–V NW arrays have unique properties that hold promise for optoelectronic applications.^1−3^ Radial strain relaxation allows for combining lattice-mismatched materials, enabling heterostructure devices not possible in planar form.^4−6^ In vertical NW arrays, nanophotonic resonance contributes to highly efficient light absorption despite the fact that only a small fraction of the surface is covered by NWs.^7,8^ This has led to considerable interest in III–V NW array photovoltaics and successful fabrication of devices achieving efficiencies exceeding 15% .^9−12^

However, the efficiency of single-junction photovoltaic devices cannot exceed the Shockley–Queisser limit.^13^ To achieve a higher efficiency, multijunction photovoltaics have been developed and set record efficiencies in the form of planar III–V devices.^14,15^ To materialize the benefits of III–V NW photovoltaics, the development of high-efficiency multijunction devices is paramount.^16,17^ One line of work is focusing on the growth of photovoltaic NWs on Si, which would open a door to a tandem configuration with a III–V NW top cell and a planar Si bottom cell.^18−20^ However, certain advantages of the III–V NW system do not materialize in such a setup. In particular, the reduced susceptibility to radiation damage observed for NW structures in theoretical as well as experimental studies is only relevant as long as all the active components are located within the NW.^21,22^ Given that photovoltaic devices for space are an important scenario of application for III–V NW array devices,^21^ this motivates the development of NW multijunction photovoltaics with multiple p–n junctions located within a NW. Previous work in our group has established parameters for the growth of axially defined GaInP/InP double-junction as well as GaInP/InP/InAsP triple-junction photovoltaic NWs, which were grown in arrays but evaluated as single NWs.^23,24^ Please note that these combinations of materials would not be possible using planar layers but are enabled by radial strain relaxation via the free surface of the NWs.^6,24^ In this paper, we report on the processing of GaInP/InP tandem-junction NW arrays into fully functional photovoltaic devices, contacting approximately 3 million NWs in parallel on each 800 × 800 μm^2^-sized device. By changing the Ga_*x*_In_1–*x*_P composition *x* between samples, we aim to understand the influence of the top cell composition on device performance. Thus, we present axially defined tandem-junction NW photovoltaic devices comprising GaInP and InP subcells located within the same NW.

## Experimental Methods

Gold (Au) seed particles were defined on an n-type InP substrate in a hexagonal pattern (pitch, 500 nm; diameter, 200 nm) using displacement Talbot lithography (DTL), E-Beam evaporation of 65 nm-thick Au, and lift-off.^25,26^ The NW growth was performed in an Aixtron 200/4 MOVPE reactor; all of the growth steps and used parameters are summarized in Tables S1 and S2. A prenucleation step at 280 °C was used to preserve the hexagonal pattern of Au particles, followed by annealing at 550 °C and NW growth at 440 °C.^27^ The precursors trimethylindium (TMIn), phosphine (PH_3_), and triethylgallium (TEGa) were used for NW growth. The NW length was monitored *in situ* using a LayTec EpiR DA UV optical reflectometry system,^28^ and the growth time of each segment was adjusted to yield the intended segment length given in Table S1, adding to a total NW length of 2300 nm. For doping, we used hydrogen sulfide (H_2_S) and diethylzinc (DEZn) precursors. Hydrogen chloride (HCl) was used to suppress undesired radial growth.^29,30^ All precursor molar fractions used are given in Tables S1 and S2.

**Figure 1:**
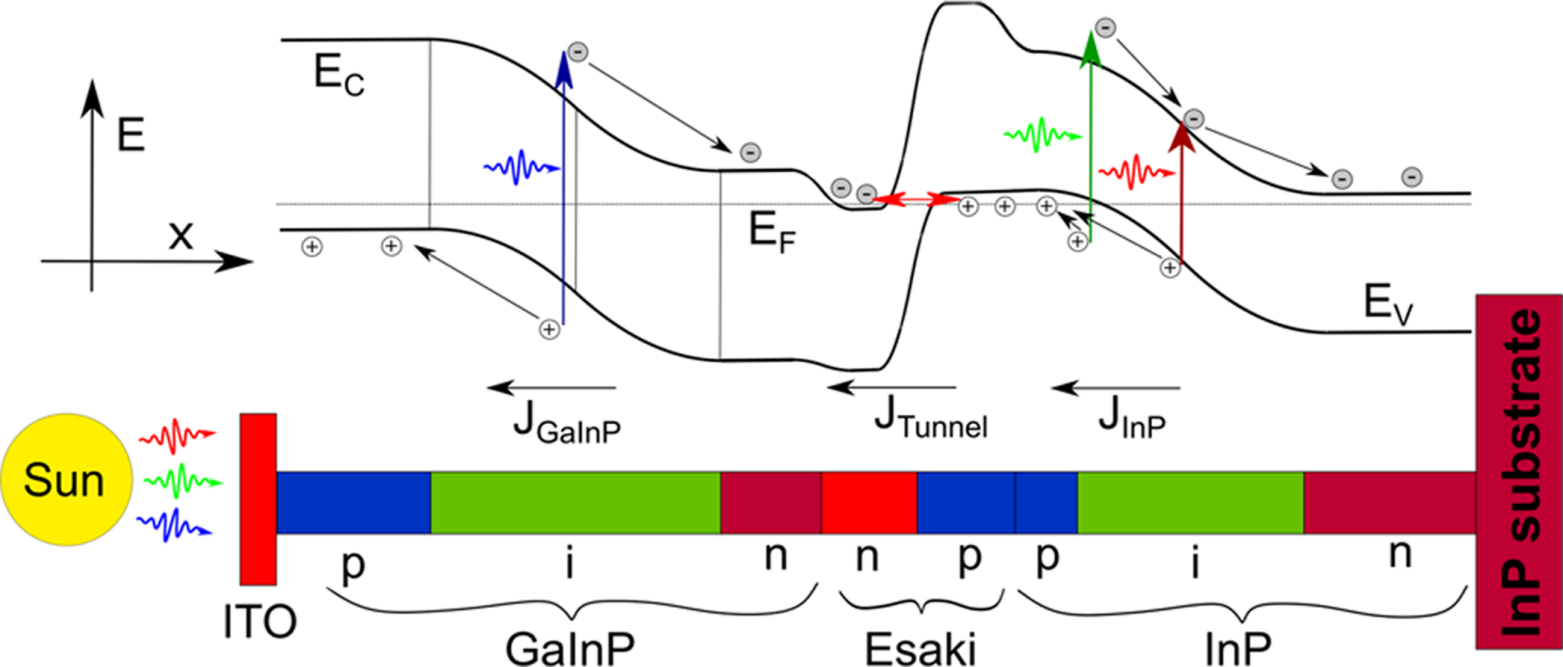
Band diagram and schematic drawing of the GaInP/InP tandem-junction nanowires. In the band diagram (top), the horizontal axis represents the position along the nanowire and the vertical axis represents energy. Absorption of light with different photon energies and tunneling through the Esaki tunnel junction are indicated. The schematic drawing (bottom) shows the segments of the tandem-junction nanowire from the n-type InP substrate (right) to the indium tin oxide (ITO) top contact (left side). The colors of the segments represent their respective doping.

The NWs consist of 3 functional parts: an InP bottom cell, an Esaki tunnel junction, and a Ga_*x*_In_1–*x*_P top cell. The Esaki tunnel diode was realized using a GaInP p^+^ and an InP n^+^ segment,^23^ and both the bottom cell (InP) and top cell (GaInP) were formed by an n–i–p junction. The NW structure and band diagram are shown schematically in Figure 1. Please note that the InP bottom cell and Esaki tunnel junction are identical across all presented samples, including the composition of the GaInP p^+^ segment, forming a part of the Esaki tunnel diode. The growth parameters during the growth of the Ga_*x*_In_1–*x*_P top cell were varied, resulting in a variation of the composition *x* between samples.

The NW arrays were processed in an analogous manner to InP NW photovoltaic devices, described in more detail in other publications and illustrated schematically in Figures S2 and S3.^31,32^ The processing steps include atomic layer deposition of SiO_*x*_, planarization using Cyclotene 3022-46 (BCB), and reactive ion etching (RIE) of excessive BCB and SiO_*x*_ covering the Au particles at the NW tips. The Au particles were wet-etched and the NW tips contacted using a 150 nm-thick sputter-coated indium tin oxide (ITO) film as a transparent front contact. Twenty-eight devices with an area of 800 × 800 μm^2^ were defined on each sample using photolithography, with separate photolithography steps for device area definition using a hard-baked S1828 frame, the ITO top contact, and Ti/Au (10 nm/200 nm) contact pads. The back contact was realized using Ti/Au (10 nm/200 nm) evaporation on the back side of the n-type InP substrate and mounting on a copper plate.

As-grown NW arrays were characterized by using X-ray diffraction (XRD) measurements performed normal to the (111)B plane. The Ga_*x*_In_1–*x*_P composition was determined using Vegard’s law based on the 2θ angle corresponding to the central value of the full width at half-maximum (fwhm) range of the respective XRD peak. Scanning electron microscopy (SEM) and electron beam-induced current (EBIC) measurements were performed in a Hitachi 8010 SEM instrument equipped with Kleindiek nanoprobes and a Point Electronic EBIC amplifier. The processed NW photovoltaic devices were characterized by using current–voltage (*I*–*V*) as well as external quantum efficiency (EQE) measurements. For this, a Cascade Microtech probe station, a G2V pico solar simulator, and a Bentham PVE300 Photovoltaic EQE setup were used.

## Results and Discussion

**Figure 2:**
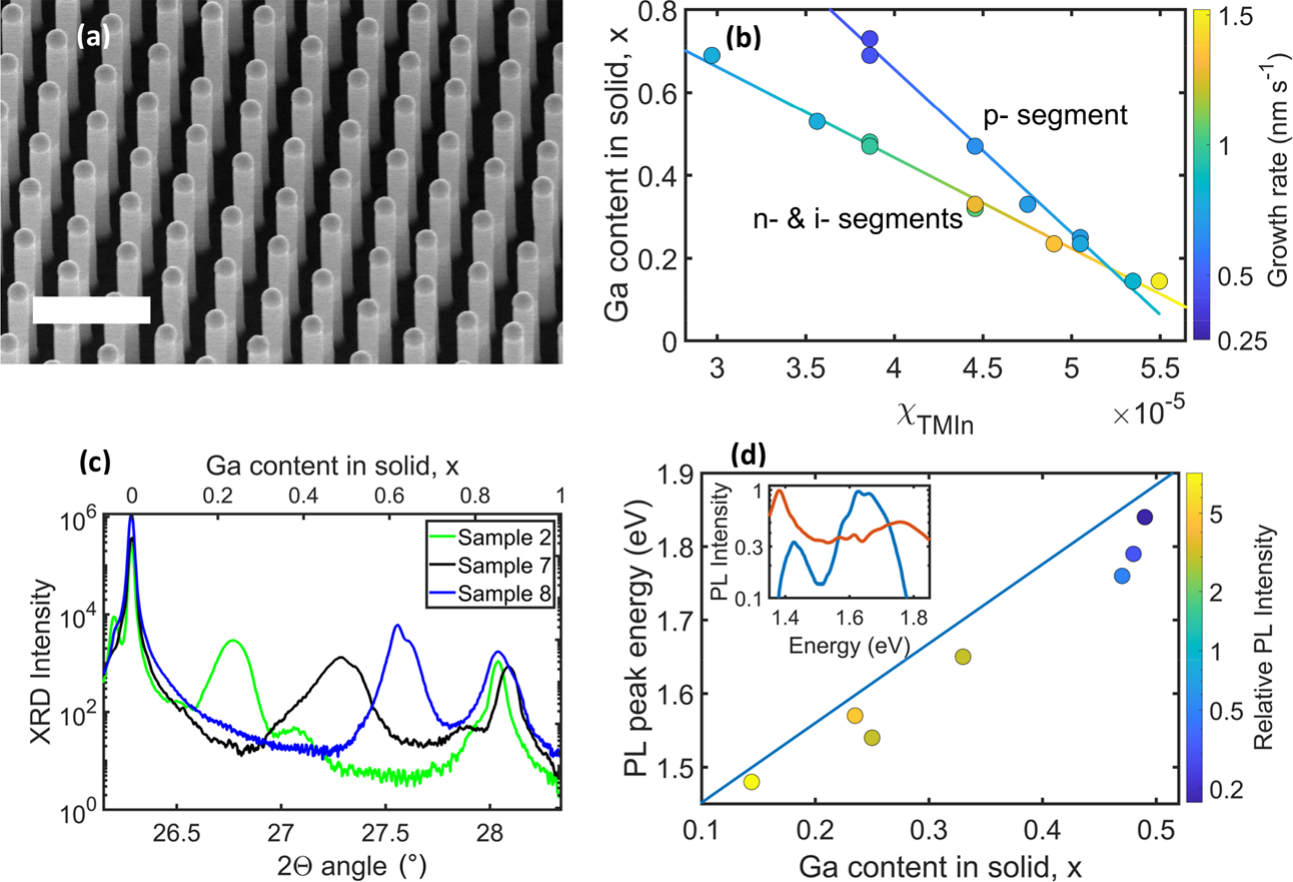
(a) SEM image of the GaInP/InP NW array of sample 7 before processing into solar cell devices. Scale bar: 1000 nm. (b) Ga content *x* in the p-type and n- and i-type GaInP segments, as determined by the XRD peak position, plotted as a function of the TMIn molar fraction used for that segment. Each data point is color-coded based on the growth rate of the respective segment. The lines represent least-square fits to the data and are in turn color-coded based on a fit of the growth rates of the segments. (c) Normalized XRD spectra of the NW arrays that were processed into devices 2, 7, and 8. The bottom *x* axis shows the measured 2Θ angle, while the top *x* axis shows the calculated Ga content *x* of Ga_*x*_In_1–*x*_P based on Bragg’s and Vegard’s laws. (d) Observed PL peak energy plotted as a function of the Ga content *x* in the n- and i-type Ga_*x*_In_1–*x*_P segments determined by the XRD peak position. Data points are color-coded using the relative PL intensity of the GaInP peak compared to that of the InP peak. The blue line represents the theoretically expected band gap energy for each GaInP composition. Inset: two representative PL spectra, normalized. Blue curve: sample 4. Red curve: sample 5.

Figure 2a shows a representative SEM image of an as-grown GaInP/InP tandem-junction NW array. XRD spectra of some processed NW arrays are shown in Figure 2c; the XRD spectra of the remaining samples are shown in Figure S1. All spectra have a common reflection at 2θ = 26.28° corresponding to the InP segments of the NWs.^33^ The GaInP segment of the Esaki tunnel diode gives rise to another common peak among all samples, seen at 2θ = 28.05°, corresponding to Ga_*x*_In_1–*x*_P with *x* = 0.86. Further, each sample has a peak corresponding to the GaInP subcell, including the n-, i-, and p-segments, which were adjusted to all have a Ga_*x*_In_1–*x*_P composition as close to each other as possible. While striving to keep the compositional variation of the GaInP subcell as small as possible, a certain variation along the NW axis is always present due to different surface diffusion properties of the In and Ga adatoms.^34^ Furthermore, the composition of the different segments is influenced by the dopants, in particular, Zn doping is known to affect the Ga_*x*_In_1–*x*_P composition.^34,35^ This effect is seen in Figure 2b, which shows the Ga_*x*_In_1–*x*_P composition *x* as a function of the TMIn molar fraction χ_TMIn_ separately for the p-segment and the n- and i-segments. For both the n- and i-segments and the p-segment, the same trends are observed: an increased growth rate and lower Ga content *x* observed in the NWs after synthesis using increased χ_TMIn_, as could be expected intuitively and in accordance with previous experimental results.^35^

We further characterized the samples by recording photoluminescence (PL) spectra of the as-grown NW arrays. Figure 2d shows the energy of the observed GaInP PL peak as a function of the composition determined by XRD. As expected, the PL peak emission energy increases with increasing Ga content *x*, following the increasing GaInP band gap. The PL peak emission energy is 0.05 ± 0.03 eV below the GaInP band gap energy obtained from the materials composition determined by XRD, which is generally to be expected because of inhomogeneous materials composition and preferential emission from low energy locations. Please note that the width of the PL peak is dominated by the compositional variation of GaInP along the NW axis. Considering the PL emission intensity, we observe a clear trend of reduced PL intensity with increasing Ga content, which can be explained by a higher nonradiative recombination rate.^36^

**Figure 3:**
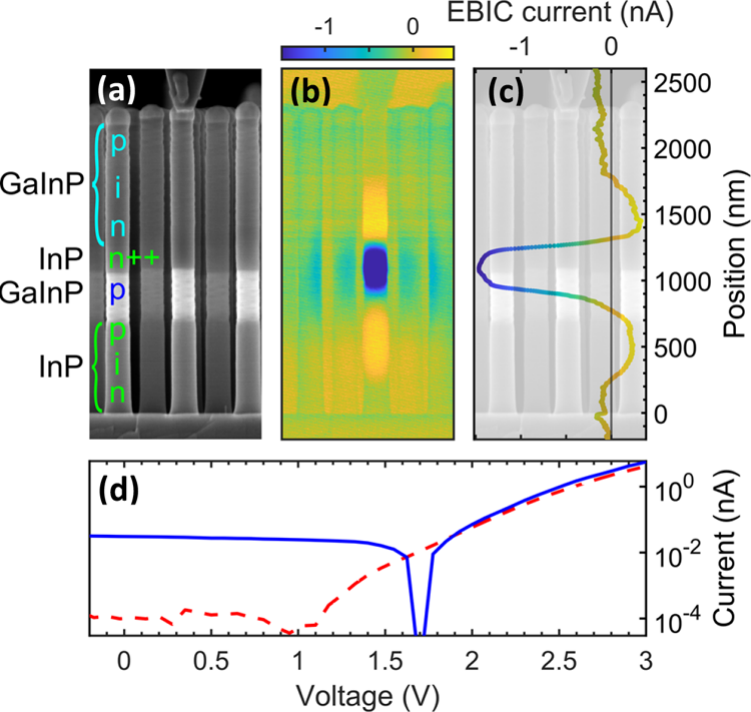
SEM and EBIC characterizations of sample 5. (a) SEM image of an NW contacted with a tip. Annotation shows the position of the bottom InP n–i–p junction, the p-GaInP/n+-InP Esaki tunnel junction, and the top GaInP n–i–p junction. (b) The EBIC image was recorded under 2.8 V forward bias. (c) EBIC line profile along the contacted NW, extracted from the image in panel (b). Overlaid with the SEM image shown in panel (a) for reference. (d) *I*–*V* curves for the same NW, recorded in the dark (red dashed line) and under electron beam illumination (blue line).

To investigate whether the functional structure on the NWs—including two n–i–p subcells connected by an Esaki tunnel diode—works as intended, we performed EBIC measurements. EBIC has previously been shown to be a valuable tool for the development and characterization of photovoltaic NWs.^23,32,37^ It is used to verify the doping profile and junction locations and, in combination with *I*–*V* measurements, study the electrical properties of the NW, including *V*_OC_ addition and the current output under illumination with the electron beam.^23,37,38^Figure 3 shows the results of EBIC measurements of an NW on a representative sample; further measurements are shown in Figure S4. As seen from Figure 3b,c, a positive EBIC current is observed when the electron beam illuminates either of the subcells, while a negative current is measured at the position of the Esaki tunnel junction under the applied forward bias conditions due to the opposite direction of the junction. These observations are in agreement with previously reported results^23^ and confirm that the doping profile of the tandem-junction photovoltaic NWs is as intended.

*I*–*V* curves for the same NW as in Figure 3a–c are shown in Figure 3d, both for dark conditions and under electron beam illumination. In the dark, the *I*–*V* curve contains a region of exponentially increasing current as a function of voltage in the forward direction, characterized by an ideality factor *n* = 6.7 ≫ 1. The ideality factor of a complex semiconductor structure can be considered as the sum of the ideality factors of the subcomponents connected in series.^39^ The ideality factors of NW diodes in many cases are close to or exceeding *n* = 2,^40,41^ rationalizing a high total ideality factor for the GaInP/InP tandem-junction device. However, the measured value of *n* = 6.7 exceeds the expected sum of the single junction ideality factors. This effect has been observed similarly for other multijunction photovoltaic NWs,^24^ and we believe that future work investigating the origin of the high ideality factor could reveal possible pathways for improving overall device performance.

Under exposure to the scanning electron beam, a photocurrent of 0.03 nA is generated, which corresponds to 14 mA/cm^2^ if multiplied with the density of the NWs in the array, 460 million NWs per cm^2^.^31^ The single-NW open-circuit voltage is measured to be *V*_OC_ = 1.7 V, a result of voltage addition from the GaInP and InP subcells, thus confirming the intended functionality of the tandem-junction device. For samples with higher Ga content *x* in the Ga_*x*_In_1–*x*_P top cell and, thus, higher GaInP band gap, an increase in *V*_OC_ is measured, reaching up to *V*_OC_ = 2.45 V for *x* = 0.69 as seen in Figure S4. Interestingly, the observed increase in single-NW *V*_OC_ exceeds the increase in the band gap; the reasons for this are currently beyond our understanding.

**Figure 4:**
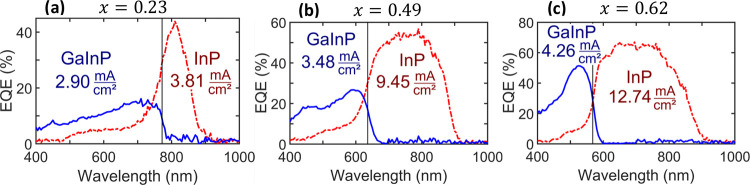
EQE characterization of processed devices on (a) sample 2, (b) sample 7, and (c) sample 8. The blue curve shows the EQE measurement performed under white light bias, corresponding to the response of the GaInP subcell. The dashed red line shows the EQE measurement under UV light bias, corresponding to the response of the InP subcell. A black vertical line marks the transition between the regions where most of the light is absorbed in the top and bottom subcells. This occurs at λ = 772 nm (*E*_*ℏ*ω_ = 1.60 eV) for sample 2, λ = 636 nm (*E*_*ℏ*ω_ = 1.95 eV) for sample 7, and λ = 568 nm (*E*_*ℏ*ω_ = 2.18 eV) for sample 8. For each subcell, the theoretically possible generated current density *J* is indicated, as calculated by integrating the EQE spectrum multiplied by the solar illumination spectrum AM1.5G.

**Figure 5:**
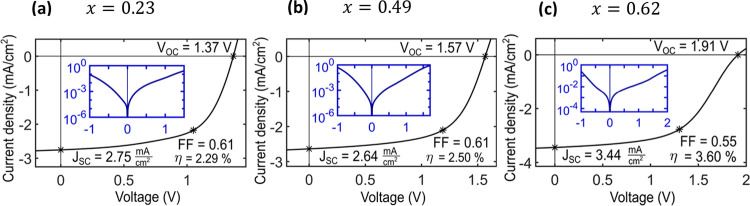
*I*–*V* characterization on processed devices on (a) sample 2, (b) sample 7, and (c) sample 8. The main figure *I*–*V* curves are measured under AM1.5G illumination. The open-circuit voltage *V*_OC_, short circuit current density *J*_SC_, fill factor FF, and device efficiency η are indicated. Inset: dark *I*–*V* curve, logarithmic *y* axis.

A total of six samples with varying Ga_*x*_In_1–*x*_P compositions in the range of *x* = 0.23–0.69 were processed into photovoltaic devices, as summarized in Table S3. The device performance characterization results for the three samples with the best performance are shown in Figures 4 and 5, and corresponding plots for the remaining three samples are shown in Figures S5 and S6.

Figure 4 shows the EQE of the GaInP and InP subcells for 3 samples with different Ga_*x*_In_1–*x*_P compositions *x*. To measure the EQE of one of the subcells connected in series in a tandem device, we have to create measurement conditions that make the respective subcell current-limiting.^42^ For measuring the EQE of the bottom InP subcell, a UV light bias (λ = 395 nm) was applied. The top GaInP subcell effectively absorbs UV light and generates a photocurrent, while the InP bottom cell is not reached by UV light. This renders the InP subcell current-limiting under these particular measurement conditions, thus allowing the EQE measurement of that subcell with the chopped light from the monochromator.^42,43^ For measuring the GaInP top subcell, white light bias illumination was used, which resulted in the GaInP top subcell becoming current-limiting due to efficient absorption of long-wavelength light by the InP subcell.

The region of high EQE of each subcell is limited by different effects toward long and short wavelengths, respectively. For long wavelength, the photon energy is only sufficient to generate electron–hole pairs starting at the energy defined by the material’s band gap. The InP subcell starts absorbing at around λ = 920 nm for all samples, roughly corresponding to the InP band gap. The onset of absorption for the Ga_*x*_In_1–*x*_P subcell depends on the band gap and, thus, the composition *x*. The onset of absorption for the GaInP top cell simultaneously also marks a decrease in the EQE of the bottom InP subcell. This is due to the reduced amount of light that reaches the bottom InP cell due to efficient absorption in the top GaInP cell. Combined, these effects lead to the presence of two wavelength regions with absorption and photocurrent generation predominantly occurring in the GaInP top cell and InP bottom cell, respectively. As seen in Figure 4, the wavelength of transition between these regions decreases with increasing Ga content *x* in GaInP, in agreement with the increasing band gap of GaInP.^44^

Under solar illumination, the integral of the EQE multiplied with the solar intensity determines the current generated by each subcell.^43^ This value was calculated by using the AM1.5G spectrum, and it is indicated in Figure 4 for both subcells. In all cases, the potential current generation of the InP subcell amounts to a higher value than for the GaInP subcell. We interpret this as a result of the higher surface recombination velocity of the GaInP NW segment compared to InP.^36^ This results in the GaInP subcell acting as the current-limiting component. Interestingly, an increased Ga content, despite the narrower wavelength range of absorption and contrary to the theoretical expectation, leads to an increased current generated by the GaInP subcell. This trend of increasing GaInP subcell performance with increasing Ga content *x*, as seen in the three presented samples in Figure 4, is likely only coincidental, as no such trend is present between the remaining three samples presented in Figure S5. It highlights, however, that the effect of variations in the quality of the GaInP subcell is larger than the change in wavelength region of absorption and that further work on understanding the causes of the GaInP subcell performance variation is necessary. Please note that the InP subcell current density follows the expected pattern, increasing with increasing GaInP band gap; however, it does not influence the overall device performance because it is not current-limiting.

In Figure 5, *I*–*V* curves of the processed tandem-junction NW photovoltaic devices are shown, measured under dark conditions as well as under AM1.5G illumination. The dark *I*–*V* curves show diode behavior, with a region of exponential current increase as a function of forward bias voltage. The ideality factor *n* ≫ 1 (ranging between 6 and 9, with the exception of sample 8, where *n* = 14) is in agreement with the measurements on single NWs shown in Figure 3 and Figure S4. At reverse bias, surprisingly large currents pass through the devices, even surpassing the forward current at the same absolute voltage. As this is not the case in the *I*–*V* curves measured on individual unprocessed NWs, it has to be associated with defects introduced during processing, which lead to a parasitic shunt path, possibly along the NW surface.

Under AM1.5G illumination, the devices show open-circuit voltages in the range of *V*_OC_ = 1.37–1.91 V as a result of the voltage addition of the subcells. For comparison, the *V*_OC_ of single-junction InP NW devices is typically in the range of *V*_OC_ = 0.5–0.8 V.^2^ The trend of increasing tandem-junction device *V*_OC_ as a function of increasing Ga content in the top junction reflects the increasing top junction band gap.

The observed short-circuit current density correlates well with the value expected from the EQE of the current-limiting GaInP subcell, albeit being slightly lower for all samples. The overall device performance for the processed NW tandem-junction photovoltaic devices reaches up to η = 3.6%, which is significantly lower than the efficiencies that have been achieved in InP single-junction NW photovoltaic devices. The reason for the low efficiency is the low short-circuit current density, *J*_SC_, which is limited by the GaInP subcell. Possible reasons for this include higher recombination rates on the GaInP NW surface,^36,45,46^ which can have a detrimental effect on NW solar cell performance. The large surface area at the NW sidewalls strongly impacts device performance because it is intersecting the active region p–i–n junction.^11,47,48^ In this work, we used a passivation scheme based on SiO_*x*_, which has been tested and optimized for InP nanowire solar cells but not GaInP nanowires.^37^ The investigation and improvement of the GaInP NW segment surface and its passivation are important pathways for future work.

Interestingly, we observed a significant difference in performance between samples 7 and 8 compared to samples 5 and 10 with similar GaInP compositions. The difference between these samples is the molar fraction of phosphine that was used, where samples 7 and 8 were grown under χ_PH_3__**=** 6.9 × 10^–3^ as compared to χ_PH_3__**=** 1.38 × 10^–2^ used for the GaInP subcell growth for the remaining samples. This has resulted in significantly higher photovoltaic device performance for samples 7 and 8, leading us to conclude that the lower phosphine molar fraction of χ_PH_3__**=** 6.9 × 10^–3^ has a beneficial effect on the GaInP subcell performance. This is in spite of group V vacancies, which are expected to form at lower phosphine molar fraction.^11^ It can be speculatively attributed to differences in the surface recombination velocity due to changes in the crystal structure forming in the NWs during synthesis at different V/III ratios.^49,50^ While we cannot reach a conclusive answer about the mechanism by which the phosphine molar flux affects device performance, this observation clearly points to changes in growth conditions as an important and sensitive lever. This motivates further studies of variations in GaInP growth parameters and their effects on the tandem-junction NW photovoltaic device performance in combination with efforts to improve surface passivation.

Due to the unexpected behavior of the GaInP subcell, an optimal value for the GaInP subcell composition, which would be determined from the condition of current matching of the subcells, cannot be established. In theory, the expected ideal GaInP composition is Ga_0.51_In_0.49_P.^51^ However, in practice, the non-ideal optoelectronic properties of either subcell can lead to a different optimal GaInP composition. This will depend, among other factors, on the quality of GaInP surface passivation. The devices we present in this work are the first tandem-junction III–V nanowire solar cells reported, and significant further efforts are needed to realize an optimized version.

## Conclusions

In summary, we have reported on GaInP/InP tandem-junction NW array photovoltaic devices. We have processed 800 × 800 μm^2^-sized devices on several samples with varying GaInP top cell composition. The resulting NW photovoltaic devices show voltage addition by GaInP and InP subcells, resulting in an open-circuit voltage of up to *V*_OC_ = 1.91 V. The EQE curves of the subcells shift as expected with varying GaInP compositions and reveal that the GaInP subcell is current-limiting for all samples. Marked performance differences between samples grown under different PH_3_ molar fractions point to a strong sensitivity toward changes in the growth conditions, possibly due to the influence on the NW sidewall surface recombination rate. Therefore, further studies are needed to map out optimal growth conditions for the GaInP subcell as well as improved surface passivation schemes. GaInP/InP tandem-junction photovoltaic devices with improved performance could become promising candidates for next-generation photovoltaics for space applications.

## Supporting Information Available

Figures presenting further XRD spectra, vertical and device processing schematics, EBIC, EQE, and *J–V* measurement results, and tables listing growth parameters, overview of samples, and detailed photovoltaic device performance characteristics (PDF)
