# Supplementary material for: Vertically Processed GaInP/InP Tandem-Junction Nanowire Solar Cells
Source: ACS Appl Nano Mater. 2024 Jan 8;7(2):2352–8. doi: 10.1021/acsanm.3c05909 (PMC10825819; doi:10.1021/acsanm.3c05909)
Supplement: Supplementary file 1 — an3c05909_si_001.pdf [file an3c05909_si_001.pdf]

Supporting Information

Vertically-Processed GaInP/InP Tandem Junction Nanowire Solar Cells

*David Alcer[†], Matteo Tirrito[†], Lukas Hrachowina[†], Magnus T. Borgström[†]**

[†] *NanoLund and Division of Solid State Physics, Lund University, Box 118, 221 00 Lund, Sweden*

* Email: magnus.borgstrom@ftf.lth.se

Figure S1 shows the XRD spectra of all GaInP/InP NW arrays that were processed into devices.

Figure S2 shows the processing steps for vertical processing of NW arrays.

Figure S3 shows the photolithography steps used for device definition.

Figure S4 shows the results of EBIC characterization of all GaInP/InP tandem junction photovoltaic NW samples that were processed into devices.

Figure S5 shows EQE measurement results for the samples not included in Figure 4 in the main text.

Figure S6 shows I-V characteristics of the samples not shown included in Figure 5 in the main text. Measurements were performed both in the dark and under AM1.5G illumination.

Table S1 summarizes the growth parameters used for the growth of all samples, detailed segment by segment.

Table S2 shows the samples that have been grown and the precursor molar fractions used for each sample. The resulting GaInP composition as determined by XRD measurements is included. The table also gives an overview about which sample is included in which figure, both in the main text and SI.

Table S3 gives an overview about photovoltaic device performance of all processed samples. Efficiency η, open-circuit voltage V_{OC}, short-circuit current density J_{SC} and fill factor FF are reported both for a selected top-performing device as well as average (and standard deviation) values of the measured devices.

Table S4 presents the photovoltaic device performance data for all measured devices on all samples. Each processed sample contained 28 devices, of which some devices were not measureable in certain cases. A number of devices were identified as outliers as marked in the table.

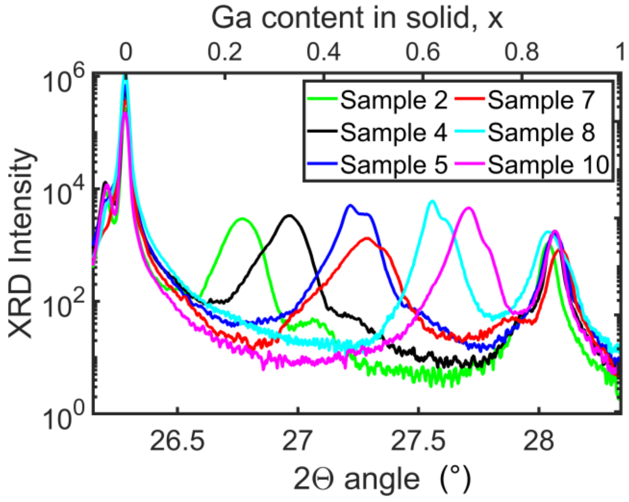

Figure S1: Normalized XRD spectra of all NW arrays that were processed into devices. The bottom x-axis shows the measured 2Θ angle, while the top x-axis shows the calculated Ga content x of the $\mathrm{Ga_xIn_{1-x}P}$, based on Bragg's and Vegard's laws.

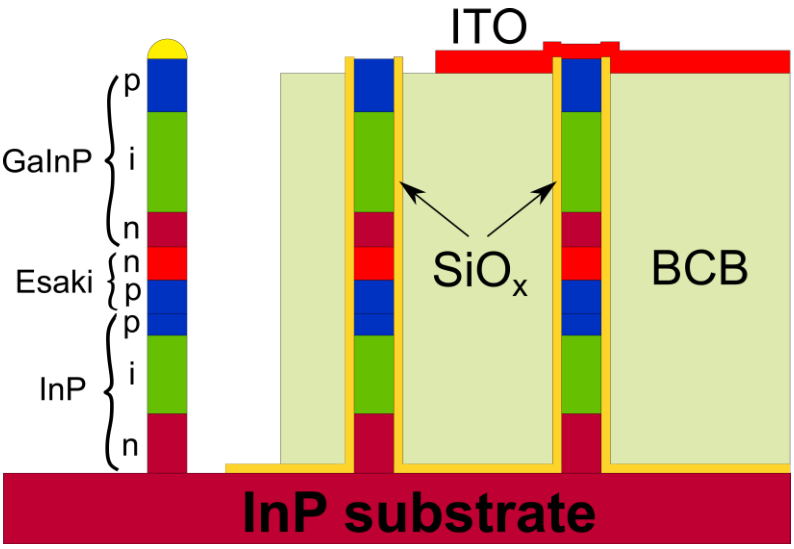

Figure S2: Schematic illustration of the processing steps used for the vertical processing of GaInP/InP nanowire arrays. SiO_x stands for non-stochiometric silicon oxide, ITO for indium tin oxide, and BCB for bisbenzocyclobutene. The color of the segments represents their respective doping.

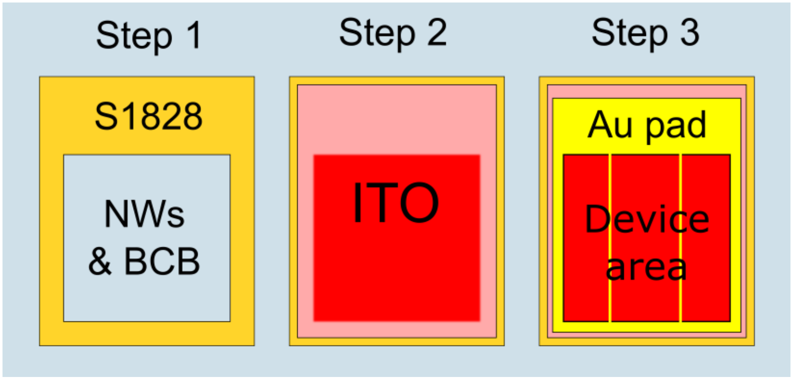

Figure S3: Schematic illustration of the photolithography steps used for the definition of tandem junction solar cell devices made from GaInP/InP nanowire arrays. S1828 is a photoresist, which is hard-baked after step 1.

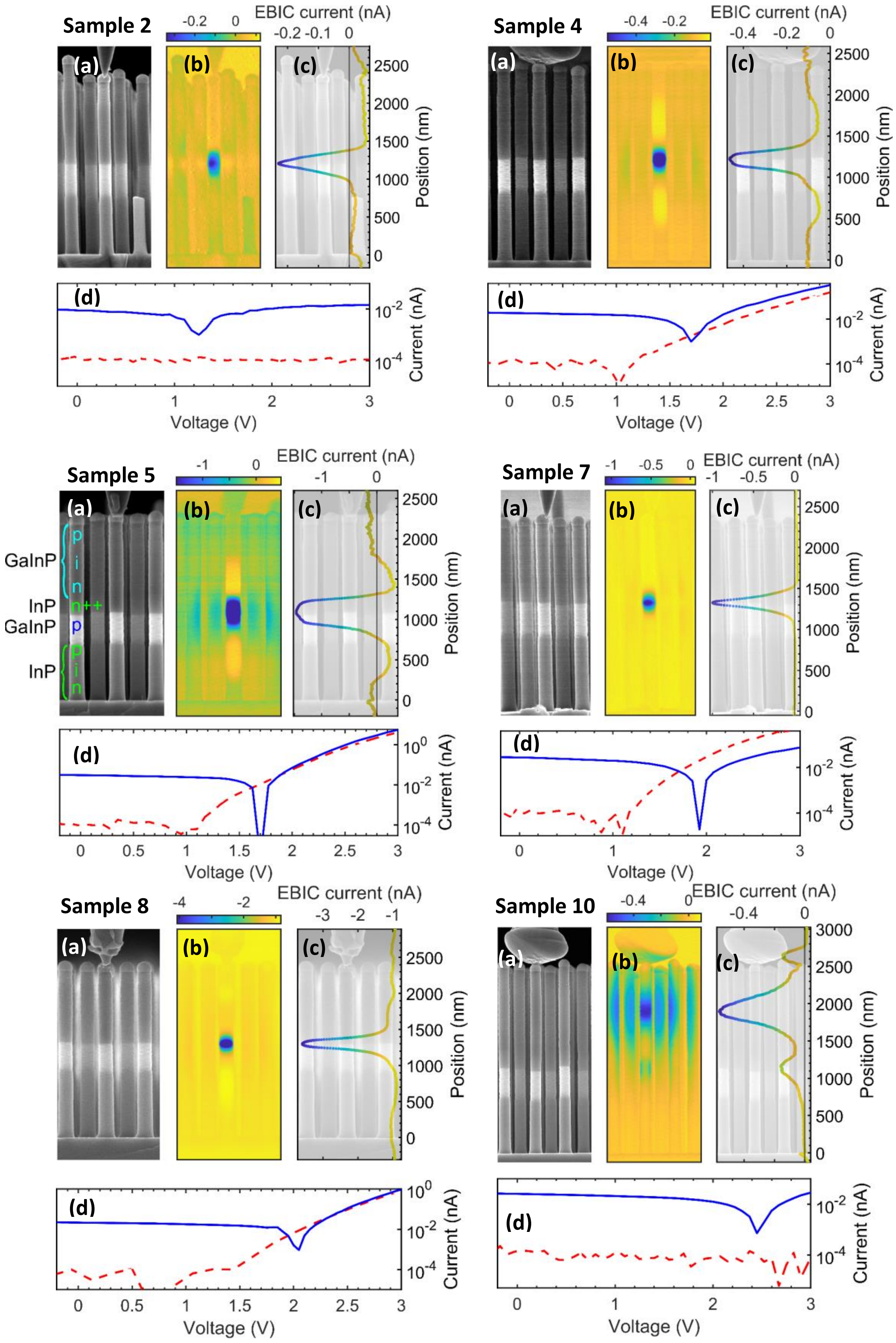

Figure S4: SEM and EBIC characterization of sample 2, sample 4, sample 5, sample 7, sample 8 and sample 10. Subpanels for each sample: **(a)** SEM image of a NW contacted with a tip. **(b)** EBIC image recorded under forward bias. **(c)** EBIC line profile along the contacted NW, extracted from the image in (b). Overlaid with the SEM image shown in (a), for reference. **(d)** IV-curves for the same NW, recorded in the dark (red dashed line) and under electron beam illumination (blue line).

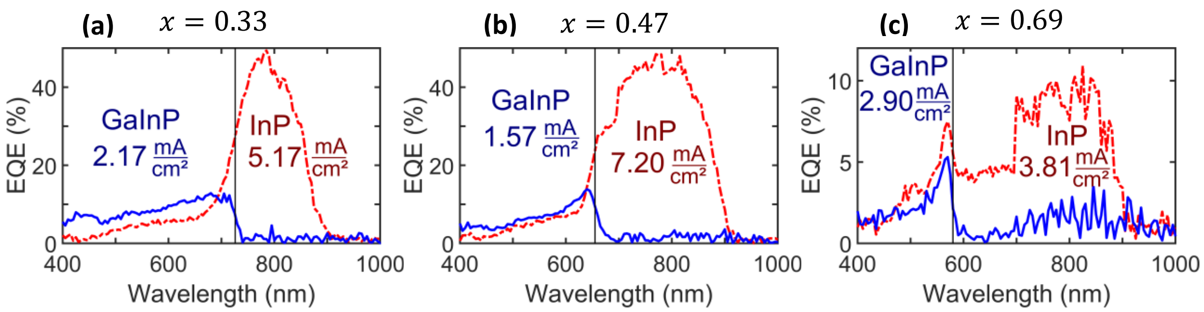

Figure S5: EQE characterization of processed devices on the samples **(a)** Sample 4 **(b)** Sample 5 and **(c)** Sample 10. The blue curve shows the EQE measurement performed under white light bias, corresponding to the response of the GaInP subcell. The dashed red line shows the EQE measurement under UV light bias, corresponding to the response of the InP subcell. A black vertical line marks the transition between the regions where current generation in the top- and bottom-subcell dominates, respectively. This occurs at $\lambda = 726$ nm for sample 4, $\lambda = 655$ nm for sample 5 and $\lambda = 580$ nm for sample 10. For each subcell, the theoretically possible generated current is indicated, as calculated by integrating the EQE spectrum multiplied with the solar illumination spectrum AM1.5G.

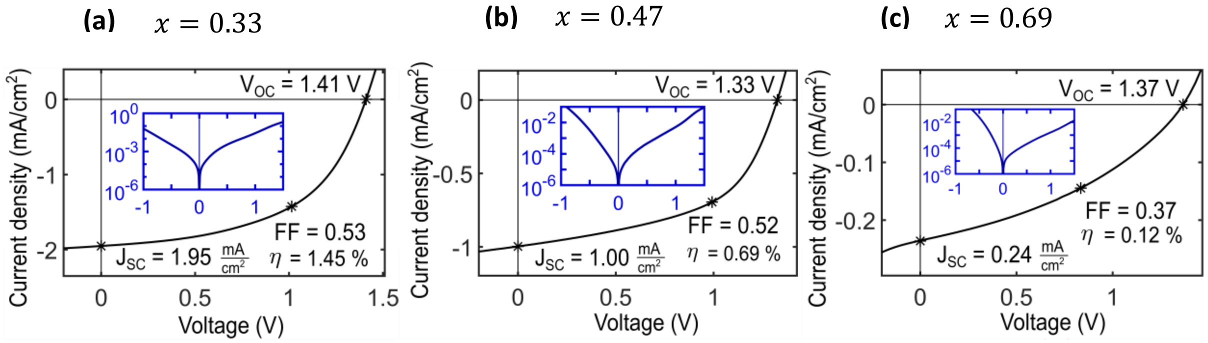

Figure S6: I-V characterization on processed devices on the samples **(a)** Sample 4 **(b)** Sample 5 and **(c)** Sample 10. The main figure I-V curves are measured under AM1.5G illumination. The open-circuit voltage V_{OC}, short circuit current density J_{SC}, fill factor FF and device efficiency η are indicated. **Inset**: Dark I-V curve, logarithmic y-axis.

Table S1: Growth parameters during the MOVPE growth of the NW arrays in an Aixtron 200/4 MOVPE reactor. Table includes growth temperature, time and molar fractions χ of the precursor gases used during the growth of each segment. The function of the segment is given, as is the segment length. Parameters kept constant during the entire growth process include the reactor pressure $p = 100$ mBar and the total flow of 13 L/min maintained by the carrier gas Hydrogen (H_2). The NW length was monitored *in-situ* using a LayTec EpiR DA UV optical reflectometry system,[28] and the growth time of each segment was adjusted to yield the intended length. The highlighted fields mark the parameters changed between the growth of different samples, the values used for each sample are given in Table S2.

Segment	Function	Temperature	χ_{TMIn}	$\chi_{\mathrm{PH_3}}$	χ_{TEGa}	χ_{HCl}	Doping	Step Duration
Pre-nucleation anneal	Preserve hexagonal pattern [27]	280 °C	8.91×10^{-5}	6.9×10^{-3}	0	0	-	60 s
Annealing step	Desorb surface oxide	550 °C	0	3.46×10^{-2}	0	0	-	600 s
InP, n (200 nm)	Part of InP bottom junction	440 °C	5.94×10^{-5}	6.9×10^{-3}	0	1.23×10^{-4}	$\chi_{\mathrm{H_2S}} = 3.11 \times 10^{-5}$	90 s
InP, i (550 nm)	Compensation −doped segment. Part of InP bottom junction	440 °C	7.43×10^{-5}	6.9×10^{-3}	0	1.23×10^{-4}	$\chi_{\mathrm{DEZn}} = 3.0 \times 10^{-8}$	240 s
InP, p (100 nm)	Part of InP bottom junction	440 °C	5.94×10^{-5}	6.9×10^{-3}	0	1.23×10^{-4}	$\chi_{\mathrm{DEZn}} = 1.11 \times 10^{-5}$	60 s
GaInP, p (250 nm)	Part of the Esaki tunnel diode	440 °C	2.97×10^{-5}	6.9×10^{-3}	5.22×10^{-5}	1.23×10^{-4}	$\chi_{\mathrm{DEZn}} = 1.11 \times 10^{-5}$	720 s
InP, n (50 nm)	Part of the Esaki tunnel diode	440 °C	2.97×10^{-5}	6.9×10^{-3}	0	1.23×10^{-4}	$\chi_{\mathrm{H_2S}} = 3.11 \times 10^{-5}$	60 s
GaInP, n (250 nm)	Part of GaInP top junction	440 °C	χ_{TMIn} − n,i	1.38×10^{-2}	5.22×10^{-5}	1.23×10^{-4}	$\chi_{\mathrm{H_2S}} = 3.0 \times 10^{-6}$	240 s - 360 s
GaInP, i (600 nm)	Undoped segment. Part of GaInP top junction	440 °C	χ_{TMIn} − n,i	1.38×10^{-2}	5.00×10^{-5}	1.23×10^{-4}		330 s - 720 s
GaInP, p (300 nm)	Part of GaInP top junction	440 °C	χ_{TMIn} − p	1.38×10^{-2}	4.18×10^{-5}	1.23×10^{-4}	$\chi_{\mathrm{DEZn}} = 1.11 \times 10^{-5}$	360 - 720 s
Cool down	Terminate growth	Reduced to 80°C	0	6.9×10^{-3}	0	0	0	

Table S2: Molar fractions χ and growth times used during the growth of samples 1 - 10, as well as the resulting $\mathrm{Ga}_x\mathrm{In}_{1-x}\mathrm{P}$ composition x in both the i,n- and p-segments, as determined by XRD. Further growth parameters are reported in Table S1. The samples that were processed into devices are highlighted, and their photovoltaic performance data is presented in Table S3 & Table S4. Sample 7 and sample 8 are marked with an asterisk because a different molar fraction of phosphine ($\chi_{\mathrm{PH_3}} = 6.9 \times 10^{-3}$) was used during the growth of these samples. The column "Figures" lists all subfigures that data from a certain sample is included in.

	χ_{TMIn} – n,i	χ_{TMIn} - p	Time, GaInP n	Time, GaInP i	Time, GaInP p	$x_{\mathrm{i,n}}$	x_{p}	Figures
Sample 1	5.50×10^{-5}	5.35×10^{-5}	240 s	330 s	360 s	0.14	$= x_{\mathrm{i,n}}$	Figure 2 (b,d)
**Sample 2**	4.90×10^{-5}	5.05×10^{-5}	270 s	360 s	390 s	0.23	$= x_{\mathrm{i,n}}$	Figure 2 (b,c,d)<br>Figure 4 (a)<br>Figure 5 (a)<br>Figure S1<br>Figure S4
Sample 3	4.46×10^{-5}	5.05×10^{-5}	330 s	480 s	420 s	0.32	0.25	Figure 2 (a,b,d)
**Sample 4**	4.46×10^{-5}	4.75×10^{-5}	300 s	360 s	420 s	0.33	$= x_{\mathrm{i,n}}$	Figure 2 (b,d,inset)<br>Figure S1<br>Figure S4<br>Figure S5 (a)<br>Figure S6 (a)
**Sample 5**	3.86×10^{-5}	4.46×10^{-5}	300 s	570 s	450 s	0.47	$= x_{\mathrm{i,n}}$	Figure 2 (a,b,d,inset)<br>Figure 3<br>Figure S1<br>Figure S5 (b)<br>Figure S6 (b)
Sample 6	3.86×10^{-5}	3.86×10^{-5}	300 s	570 s	510 s	0.48	0.73	Figure 2 (a,b,d)
**Sample 7*** $\chi_{PH_3} = 6.9 \times 10^{-3}$	3.86×10^{-5}	3.86×10^{-5}	360 s	720 s	720 s	0.49 *	$= x_{\mathrm{i,n}}$ *	Figure 2 (c,d)<br>Figure 4 (b)<br>Figure 5 (b)<br>Figure S1<br>Figure S4
**Sample 8*** $\chi_{PH_3} = 6.9 \times 10^{-3}$	2.97×10^{-5}	2.97×10^{-5}	360 s	720 s	720 s	0.62 *	$= x_{\mathrm{i,n}}$ *	Figure 2 (c)<br>Figure 4 (c)<br>Figure 5 (c)<br>Figure S1<br>Figure S4
Sample 9	3.57×10^{-5}	3.86×10^{-5}	360 s	720 s	720 s	0.53	0.73	Figure 2 (a,b)
**Sample 10**	2.97×10^{-5}	3.86×10^{-5}	360 s	720 s	660 s	0.69	$= x_{\mathrm{i,n}}$	Figure 2 (b)<br>Figure S4<br>Figure S5 (c)<br>Figure S6 (c)

Table S3: Photovoltaic device preformance results for the processed samples. Efficiency η, open-circuit voltage V_{OC}, short-circuit current density J_{SC} and fill factor FF are reported. Values for a selected top-performing device as well as average values and standard deviations (S.D.) for all measured devices (after removing outliers) are given. The full dataset including measurement results of all devices is compiled in Table S4. All parameters were measured under AM1.5G illumination. Sample 7 and sample 8 are marked with an asterisk because a different molar fraction of phosphine ($\chi_{PH_3} = 6.9 \times 10^{-3}$) was used during the growth of these samples.

	x in $Ga_xIn_{1-x}P$	η (%)	V_{OC} (V)	J_{SC} (mA/cm^2)	FF	η mean (S.D.) (%)	V_{OC} mean (S.D.) (V)	J_{SC} mean (S.D.) (mA/cm^2)	FF mean (S.D.)
Sample 2	0.23	2.29	1.37	2.75	0.61	1.91 (0.23)	1.31 (0.03)	2.59 (0.18)	0.56 (0.04)
Sample 4	0.33	1.45	1.42	1.95	0.53	1.18 (0.18)	1.33 (0.07)	1.87 (0.16)	0.47 (0.04)
Sample 5	0.47	0.69	1.33	1.00	0.52	0.64 (0.06)	1.27 (0.03)	0.94 (0.08)	0.53 (0.02)
Sample 7*	0.49	2.50	1.57	2.64	0.61	2.27 (0.24)	1.48 (0.04)	2.51 (0.28)	0.61 (0.01)
Sample 8*	0.62	3.60	1.91	3.44	0.55	2.92 (0.44)	1.83 (0.07)	2.83 (0.45)	0.56 (0.03)
Sample 10	0.69	0.12	1.37	0.24	0.37	0.09 (0.02)	1.16 (0.11)	0.20 (0.03)	0.37 (0.01)

Table S4: Photovoltaic device preformance results for the processed samples. Each sample contains 28 devices with a size of $800 \times 800\ \mu m^2$ each, of which some devices can be nonfunctional due to processing issues, marked with '-'. Efficiency η, open-circuit voltage V_{OC}, short-circuit current density J_{SC} and fill factor FF are reported. Values for all measured devices are given, sorted in descending order of efficiency η. Results for devices that are considered outliers are marked in red, these are not included in the calculation of average performance results (and standard deviations) shown in Table S3. A selected top performance device, highlighted in green, is reported on in Figure 4, Figure 5, Figure S5 & Figure S6 as well as Table S3. All parameters were measured under AM1.5G illumination. Sample 7 and sample 8 are marked with an asterisk because a different molar fraction of phosphine ($\chi_{PH_3} = 6.9 \times 10^{-3}$) was used during the growth of these samples. For sample 8, only 14 devices were measureable because the sample broke during processing.

η (%)	mean	S.D.	1	2	3	4	5	6	7	8	9	10	11	12	13	14	15	16	17	18	19	20	21	22	23	24	25	26	27	28
Sample 2	1.91	0.23	2.29	2.24	2.21	2.20	2.18	2.13	2.06	2.04	2.02	1.99	1.97	1.95	1.94	1.92	1.86	1.84	1.79	1.74	1.69	1.66	1.65	1.65	1.59	1.56	1.55	1.16	1.10	0.44
Sample 4	1.18	0.18	1.45	1.44	1.42	1.35	1.34	1.29	1.28	1.27	1.25	1.22	1.18	1.16	1.15	1.14	1.14	1.13	1.09	1.05	0.95	0.88	0.87	0.83	0.78	0.67	0.05	0.04	0.01	0.01
Sample 5	0.64	0.06	1.14	0.84	0.73	0.72	0.70	0.69	0.69	0.69	0.69	0.67	0.66	0.66	0.64	0.62	0.62	0.60	0.59	0.58	0.58	0.57	0.56	0.55	0.46	0.43	0.39	0.22	0.11	-
Sample 7*	2.27	0.24	2.89	2.73	2.58	2.50	2.47	2.41	2.39	2.37	2.36	2.35	2.32	2.31	2.28	2.25	2.23	2.21	2.15	2.14	2.13	2.11	2.10	2.10	2.07	2.02	2.02	1.96	1.77	-
Sample 8*	2.92	0.44	3.60	3.32	3.03	2.93	2.89	2.87	2.68	2.51	2.41	2.24	0.91	0.05	0.05	0.05	-	-	-	-	-	-	-	-	-	-	-	-	-	-
Sample 10	0.09	0.02	0.18	0.14	0.12	0.11	0.11	0.11	0.11	0.10	0.10	0.10	0.09	0.09	0.09	0.08	0.08	0.08	0.08	0.07	0.07	0.07	0.07	0.07	0.05	0.05	0.05	0.02	0.02	-

V_{OC} (V)	mean	S.D.	1	2	3	4	5	6	7	8	9	10	11	12	13	14	15	16	17	18	19	20	21	22	23	24	25	26	27	28
Sample 2	1.31	0.03	1.37	1.31	1.30	1.28	1.32	1.31	1.28	1.32	1.29	1.38	1.25	1.29	1.31	1.32	1.30	1.31	1.29	1.32	1.32	1.30	1.32	1.31	1.33	1.31	1.27	1.20	1.13	0.61
Sample 4	1.33	0.07	1.41	1.39	1.40	1.35	1.39	1.36	1.32	1.42	1.36	1.37	1.38	1.30	1.35	1.33	1.40	1.35	1.26	1.21	1.27	1.19	1.28	1.26	0.97	0.99	0.11	0.07	0.03	0.03
Sample 5	1.27	0.03	1.28	1.27	1.24	1.26	1.25	1.29	1.26	1.33	1.25	1.26	1.28	1.28	1.22	1.34	1.23	1.27	1.23	1.32	1.32	1.25	1.25	1.30	1.17	1.18	1.03	0.55	0.39	-
Sample 7*	1.48	0.04	1.46	1.46	1.43	1.57	1.45	1.49	1.46	1.50	1.50	1.52	1.56	1.49	1.47	1.44	1.50	1.41	1.50	1.54	1.55	1.50	1.48	1.40	1.49	1.47	1.46	1.43	1.48	-
Sample 8*	1.83	0.07	1.91	1.92	1.78	1.25	1.79	1.36	1.88	1.79	1.77	1.64	1.80	0.01	0.01	0.02	-	-	-	-	-	-	-	-	-	-	-	-	-	-
Sample 10	1.16	0.11	1.16	1.18	1.37	1.36	1.27	0.87	1.24	1.18	1.19	1.23	1.20	0.84	1.23	1.14	1.16	1.19	1.13	1.01	0.99	1.04	1.08	1.01	1.02	1.11	0.68	0.47	0.47	-

J_{SC} (mA/cm²)	mean	S.D.	1	2	3	4	5	6	7	8	9	10	11	12	13	14	15	16	17	18	19	20	21	22	23	24	25	26	27	28
Sample 2	2.59	0.18	2.75	2.88	2.78	2.82	2.79	2.64	2.78	2.52	2.53	2.41	2.74	2.70	2.65	2.74	2.72	2.55	2.47	2.50	2.49	2.42	2.35	2.58	2.28	2.33	2.27	2.08	2.12	2.06
Sample 4	1.87	0.16	1.95	1.92	1.92	2.15	2.04	1.98	2.17	1.72	1.77	1.92	1.90	1.87	1.89	1.91	1.72	1.65	1.92	1.99	1.74	1.88	1.58	1.59	1.98	1.64	1.76	2.02	1.66	1.53
Sample 5	0.94	0.08	1.70	1.30	1.07	1.08	1.02	0.98	1.02	1.00	0.98	0.98	0.95	0.96	1.00	0.89	0.95	0.88	0.87	0.84	0.84	0.83	0.84	0.88	1.08	0.92	1.00	1.14	0.98	-
Sample 7*	2.51	0.28	3.21	3.05	2.97	2.64	2.77	2.60	2.67	2.64	2.60	2.59	2.50	2.51	2.55	2.56	2.37	2.60	2.34	2.27	2.29	2.29	2.32	2.49	2.24	2.22	2.26	2.33	1.93	-
Sample 8*	2.83	0.45	3.44	3.39	2.94	5.80	2.74	5.73	2.49	2.45	2.36	2.86	0.90	14.5	14.3	8.90	-	-	-	-	-	-	-	-	-	-	-	-	-	-
Sample 10	0.20	0.03	0.41	0.31	0.24	0.22	0.24	0.39	0.24	0.23	0.23	0.21	0.21	0.33	0.19	0.19	0.19	0.19	0.18	0.20	0.20	0.17	0.16	0.17	0.15	0.13	0.20	0.14	0.15	-

FF	mean	S.D.	1	2	3	4	5	6	7	8	9	10	11	12	13	14	15	16	17	18	19	20	21	22	23	24	25	26	27	28
Sample 2	0.56	0.04	0.61	0.59	0.61	0.61	0.59	0.62	0.58	0.61	0.62	0.60	0.57	0.56	0.56	0.53	0.53	0.55	0.56	0.53	0.51	0.53	0.53	0.49	0.53	0.51	0.54	0.47	0.46	0.35
Sample 4	0.47	0.04	0.53	0.54	0.53	0.47	0.47	0.48	0.45	0.52	0.52	0.47	0.45	0.48	0.45	0.45	0.47	0.51	0.45	0.44	0.43	0.39	0.43	0.42	0.41	0.41	0.25	0.25	0.25	0.25
Sample 5	0.53	0.02	0.52	0.51	0.55	0.53	0.55	0.55	0.54	0.52	0.56	0.54	0.54	0.54	0.53	0.53	0.53	0.54	0.55	0.52	0.52	0.54	0.53	0.48	0.37	0.40	0.38	0.35	0.30	-
Sample 7*	0.61	0.01	0.62	0.61	0.61	0.61	0.61	0.62	0.61	0.60	0.60	0.60	0.59	0.62	0.61	0.61	0.63	0.60	0.61	0.61	0.60	0.61	0.61	0.60	0.62	0.62	0.61	0.59	0.62	-
Sample 8*	0.56	0.03	0.55	0.51	0.58	0.40	0.59	0.37	0.57	0.57	0.58	0.48	0.56	0.25	0.25	0.25	-	-	-	-	-	-	-	-	-	-	-	-	-	-
Sample 10	0.37	0.01	0.37	0.37	0.37	0.39	0.37	0.33	0.37	0.37	0.37	0.37	0.37	0.33	0.37	0.38	0.37	0.36	0.37	0.35	0.35	0.37	0.37	0.37	0.37	0.36	0.37	0.39	0.31	-
